# Phytochemisty and Spermatogenic Potentials of Aqueous Extract of Cissus populnea (Guill. and Per) Stem Bark

**DOI:** 10.1100/tsw.2006.343

**Published:** 2006-06-21

**Authors:** Anthony B. Ojekale, Oladipupo A. Lawal, Adedoyin K. Lasisi, Tajudeen I. Adeleke

**Affiliations:** ^1^ Department of Biochemistry, Lagos State University, Ojo, Apapa, Nigeria; ^2^ Department of Chemistry, Lagos State University, Ojo, P.M.B.1087, Apapa, Nigeria; ^3^ Department of Pharmacognosy, College of Medicine, University of Lagos, Idi- Araba, Lagos, Nigeria

**Keywords:** spermatogeny, phytochemistry, Cissus populnea, Lagos

## Abstract

*In vivo* clinical trials involving the administration of crude extracts of Cissus populnea to male subjects (normospermic, oligospermic, and azoopermic) in a 72-day study revealed that continuous exposure of the subjects to the extracts over this period did not significantly (p ≤ 0.05) alter sperm count, morphology, motility, or volume. Antimicrobial screening of the extract against some selected microbial isolates secondarily implicated in male infertility revealed total inactivity against the microbial isolates screened, i.e., *Staphylococcus aureus*, *Salmonella paratyphi*, *Escherichia coli*, *Proteus mirabilis*, *Pseudomonas aeruginosa*, *Candida albicans*, and *Klebsiella* sp. Phytochemistry revealed the presence of tannins, flavonoids, saponins, and steroids. The presence of these secondary metabolites was confirmed by thin layer chromatography. We conclude that oral administration of aqueous extracts of the stem bark of Cisssus populnea over a 72-day period to human subjects apparently had no fertility enhancement effects on sperm parameters monitored in this study.

## INTRODUCTION

It has long been demonstrated that semen quality and sperm output are frequently lower than is generally accepted among fertile men. A malfunction/arrest of spermatogenesis, ductal obstruction, hypogonadotropic hypogonadism, among others are some of the causative factors of male infertility[1]. About 20% of childless marriages result from fertility problems/sterility on the part of the male partners, while contributing to about 30–50% of others[1,2].

In Nigeria today, even though statistics are not accurate, clinical experience and focal reports dating back about a decade suggest a high male contribution, ranging between 40–51%, in childless marriages[3]. The rise in childlessness among married couples in Nigeria is further compounded by cultural, religious, and traditional beliefs. This belief system makes it quite incomprehensible for a man to come to terms with the possibility of his being infertile/sterile, as these dysfunctions are seen mainly as feminine problems.

The classical approach to therapy of male infertility is via spermatogenic drugs such as Clomiphene (Clomid). However, prescription drugs like these are usually expensive, coupled with the possibility of adulteration of such. The gradual shift to herbal therapy with its attendant increasing acceptance, even among the elites, make the herbal practitioners lay claims to having the cure to a myriad of ailments, including male infertility, irrespective of the etiology of such diseases.

An ethnobiological survey of spermatogenic plants in Nigeria reports such spermatogenic plants as *Lonchocarpus cyanescens*, *Cissus zombensis*, *Bompax buonopozense,* and *Kigelia africana*[4]. *C. populnea* extracts have been credited with antibacterial properties[5], as a component of an antisickling Nigerian herbal formula[6], as a source of gum powder, and as an antitrypanosomal plant[7].

The objective of the present study was to evaluate/find justification in the purported spermatogenic potentials of the stem bark extract of *C. populnea* via *in-vivo* clinical trials, antimicrobial screening against fertility implicating microbes, and comprehensive phytochemistry of the extracts.

## METHODS

Fresh stem bark samples of *C. populnea* were obtained by commissioning a local herbal practitioner in Oyingbo, Lagos State, in southwestern Nigeria. They were authenticated at the Department of Pharmacognosy, College of Medicine of the University of Lagos, Nigeria (PCGLH-370). The bark samples were chopped into tiny bits, rinsed thoroughly, and then blotted. The fresh, blotted, weighed samples were steeped in 1 l of sterile distilled water (23 g/100 ml water) for 72 h. The resulting crude extract was refrigerated (4°C) until needed.

The extracts were confirmed nontoxic as they were tolerated in doses as high as 200 g/kg body weight using the method of Okochi et al.[8].

A total of 24 interested, consenting, healthy male subjects from Lagos Metropolis, with recent sperm analysis results, were recruited for the study with the expressed consent of their physicians. The age range was 19–30 years. Subjects were advised to abstain from all types of drugs 10 weeks prior and throughout the duration of the study except in cases as advised by a physician. Subjects were also to abstain from sex at least 48 h before sample collection. Subjects were then divided into groups according to their pretest semen analyses, viz normospermic, oligospermic, azospermic, and the control group, respectively:

- Group I (control): Normal male, no extract was administered for the 72 days. Semen sample collected every 14 days.
- Group II (normospermic): Normal male, extract was orally administered every day for duration of study. Semen sample collected every 14 days.
- Group III (oligospermic): Extract was orally administered every day for duration of study. Semen sample collected every 14 days.
- Group IV (azoospermic): Extract was orally administered every day for duration of study. Semen sample collected every 14 days.

Standard procedures were used for semen analysis within 2 h of collection[9]. Standard phytochemistry techniques were employed to analyze plant extract[10,11,12,13,14].

All crude extracts were chromatographed on silica gel plates (G254) with the appropriate mobile phases after proper sample preparation[15]. The pathogens used in this study, i.e., *Staphylococcus aureus, Escherichia coli, Salmonella typhimurium, Saccharomycese cerevisae,* and *Candida albicans* were obtained from the Microbiology Section of Evans Pharmaceuticals, Agbara, Ogun State. Nigeria. The methods used were Agar diffusion well method of Bauer[16], and Agar diffusion disc method of Cichewa and Thorpe[17].

## Data Analyses

Data values used for plotting graphs were means, with n = 6 for all groups, while graphs were plotted using SPSS for Windows (Version 11). The same software was used in the determination of the levels of significance of the treatment regime to the parameters measured (ANOVA).

## RESULTS AND DISCUSSION

Aqueous stem bark extracts of *Cissus populnea* are widely used among the local folks, especially in the southwestern part of Nigeria as an aphrodisiac/fertility formula. The screening of the extract carried out against some selected microbes secondarily associated with male infertility in this study revealed total inactivity against all the microbes (see Table 1) contrary to the report of Kone et al.[5], who reported that the ethanolic extract of the plant was active against *E. coli, Pseudomonas aeruginosa, Staphylococcus aureus, S. pyogenes,* and *Bacillus subtilis.*

**TABLE 1**
**Antimicrobial Screening of *C. populnea* Extracts Against Some Selected Microorganisms**

| | Extracts | |
|---|---|---|
| **Parameter** | ***C. populnea*** | **Distilled Water** |
| *Staphylococcus aureus* | — | — |
| *Salmonella paratyphi* | — | — |
| *Escherichia coli* | — | — |
| *Proteus mirabilis* | — | — |
| *Pseudomonas aeruginosa* | — | — |
| *Candida albicans* | — | — |
| *Klebsiella* sp. | — | — |

—, No inhibition noticed.

The outcome of the clinical trials revealed that at the dose administered (150g/kg body weight), there was no significant ($p \leq 0.05$) difference in the levels of the parameters scored for, viz sperm count, motility, morphology, and volume (see Figs. 1–4).

The stem bark of the plant contains such secondary metabolites as tannins, flavonoids, saponins, and steroids (see Table 2), some of which in their group, members have been associated with functions related to fertility enhancement potentials[18,19,20].

The available literature on this plant to date has no report of the plant having any effect on fertility in either gender. Moody et al.[6] established the antisickling capacity of a Nigerian herbal formula containing *C. populnea*, while Geidam et al.[21] established that treatment of animals with the extract did not result in an elevation of enzymes used as liver function markers, even in diabetes-induced animals. In the Republic of Benin, the extract is used for diuresis, while the root extract is used in “cooling off” pregnant women[22].

The outcome of the present study has established the stem bark extract of the plant *C. populnea* as having secondary metabolites that possess medical potentials, but no fertility actions have been uncovered, even though the literature indicates otherwise. This may be due to the possibility of the herbal extract in use by the folks as being a polyherbal formula instead of a monoherbal formulation as worked on in this paper.

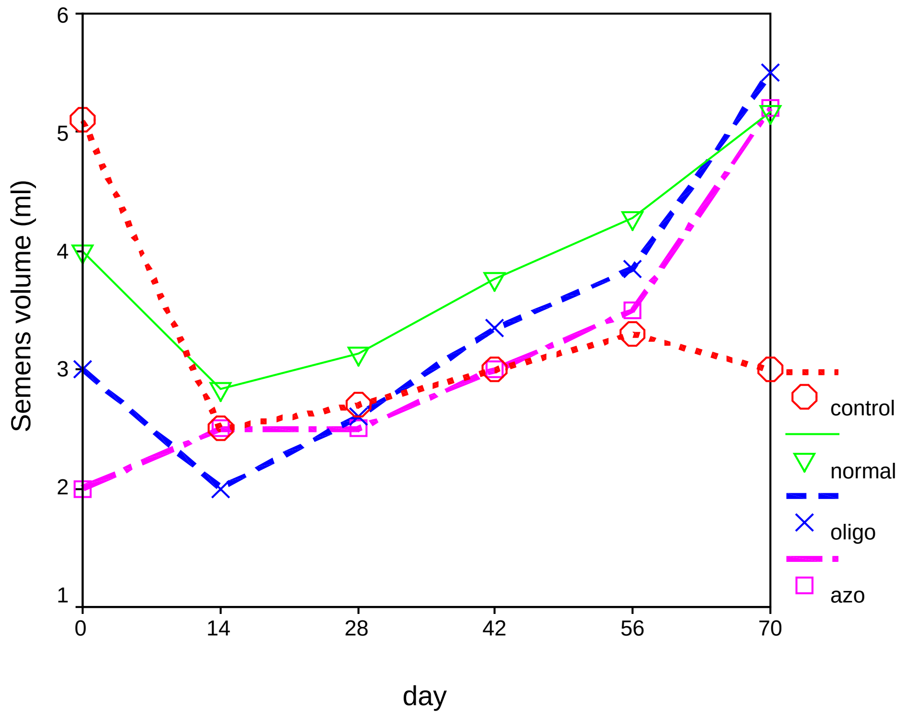

**FIGURE 1.** Effect of *C. populnea* aqueous extract on semen volume.

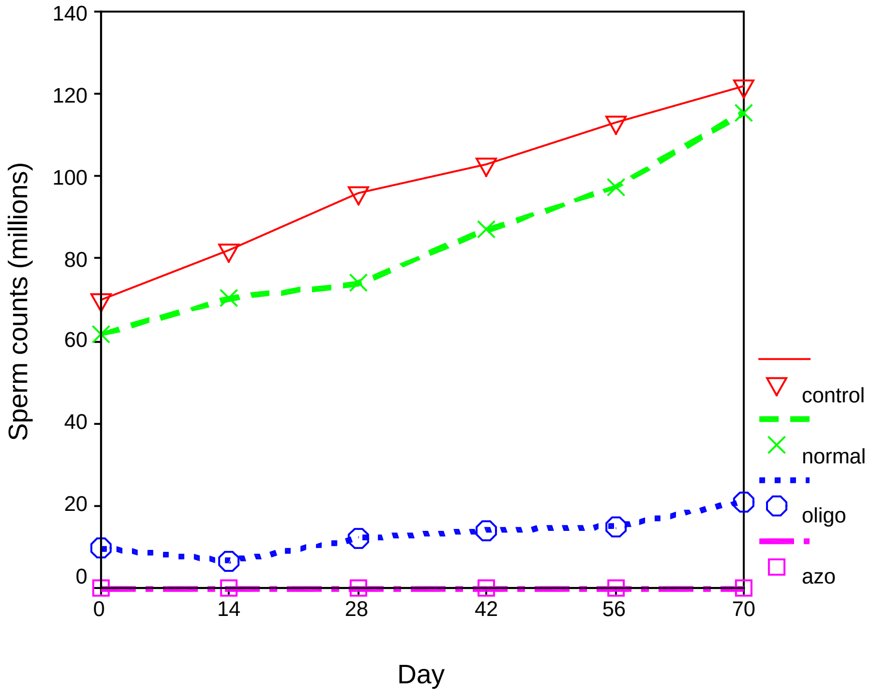

**FIGURE 2.** Effect of *C. populnea* aqueous extract on sperm count.

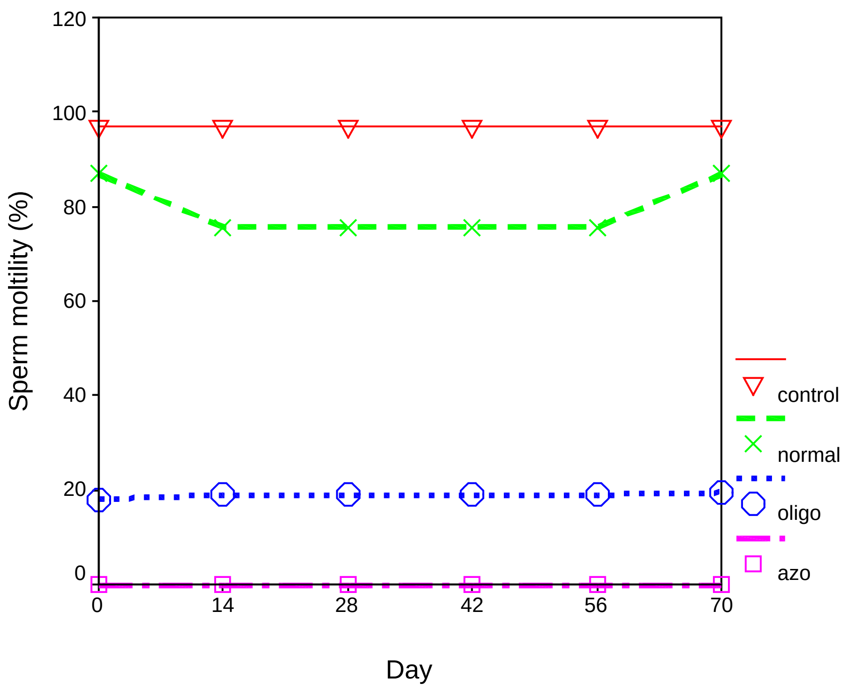

**FIGURE 3**. Effect of *C. populnea* aqueous extract on sperm motility.

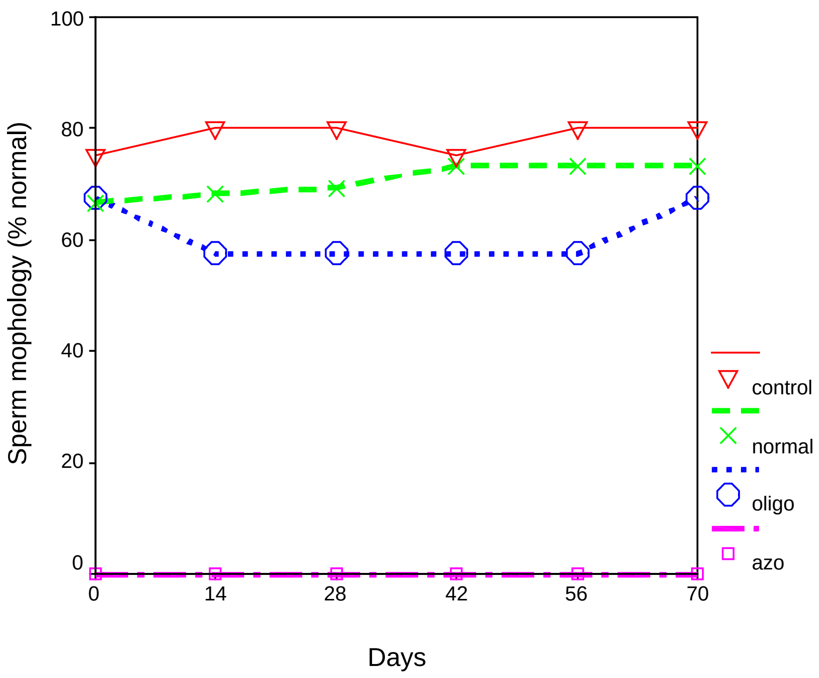

**FIGURE 4.** Effect of *C. populnea* aqueous extract on sperm morphology.

**TABLE 2**
**Phytochemistry of Stem Bark of *C. populnea***

| Parameter | Observation |
|---|---|
| Alkaloids | Absent |
| Saponins | Present |
| Flavonoids | Present |
| Tannins | Present |
| Phenolics | Absent |
| Steroidal nucleus | Present |
| Free anthraquinones | Absent |

## BIOSKETCHES

**Anthony Babajide Ojekale**, **BSc, MSc** (Biochemistry), is currently a lecturer in the Department of Biochemistry, Faculty of Science, Lagos State University, Ojo, Lagos, Nigeria. He is actively involved in scientific evaluation of medicinal plants/ formulations local to Nigeria, and is currently undergoing a PhD program, working on alternate therapy (medicinal plants) to tropical diseases. E-mail: tonyojekale@yahoo.co.uk

**Oladipupo Adejumobi Lawal**, **BSc, MSc** (Chemistry), is currently a lecturer in the Department of Chemistry, Faculty of Science, Lagos State University, Ojo, Lagos, Nigeria. He is doing a PhD program at the University of Kwa-Zulu-Natal, South Africa. His research interest is on the environment and nano-organic compounds. E-mail: ladilawal@hotmail.com

**Adedoyin Kehinde Lasisi, BSc** (Biochemistry), is an avid believer in alternate medical therapies (natural plant remedies). He is currently working on evalution of local medicinal plants and environmental matters.

**Tajudeen Isaac Adeleke, BSc** (Pharmacology/Physiology), is a laboratory technologist with the Department of Pharmacognosy of the College of Medicine of the University of Lagos, Nigeria. He is actively involved in the practice of local herbal medicine, and also in its scientific evaluation/applications.
